# *In vitro* Increased Respiratory Activity of Selected Oral Bacteria May Explain Competitive and Collaborative Interactions in the Oral Microbiome

**DOI:** 10.3389/fcimb.2017.00235

**Published:** 2017-06-07

**Authors:** Emma Hernandez-Sanabria, Vera Slomka, Esteban R. Herrero, Frederiek-Maarten Kerckhof, Lynette Zaidel, Wim Teughels, Nico Boon

**Affiliations:** ^1^Center for Microbial Ecology and Technology, Ghent UniversityGhent, Belgium; ^2^Department of Oral Health SciencesKU Leuven, Leuven, Belgium; ^3^Colgate-Palmolive Technology CenterPiscataway, NJ, United States

**Keywords:** oral pathobionts, health-associated bacteria, commensal bacteria, phenotype microarray, respiratory activity, multivariate statistical analysis

## Abstract

Understanding the driving forces behind the shifts in the ecological balance of the oral microbiota will become essential for the future management and treatment of periodontitis. As the use of competitive approaches for modulating bacterial outgrowth is unexplored in the oral ecosystem, our study aimed to investigate both the associations among groups of functional compounds and the impact of individual substrates on selected members of the oral microbiome. We employed the Phenotype Microarray high-throughput technology to analyse the microbial cellular phenotypes of 15 oral bacteria. Multivariate statistical analysis was used to detect respiratory activity triggers and to assess similar metabolic activities. Carbon and nitrogen were relevant for the respiration of health-associated bacteria, explaining competitive interactions when grown in biofilms. Carbon, nitrogen, and peptides tended to decrease the respiratory activity of all pathobionts, but not significantly. None of the evaluated compounds significantly increased activity of pathobionts at both 24 and 48 h. Additionally, metabolite requirements of pathobionts were dissimilar, suggesting that collective modulation of their respiratory activity may be challenging. Flow cytometry indicated that the metabolic activity detected in the Biolog plates may not be a direct result of the number of bacterial cells. In addition, damage to the cell membrane may not influence overall respiratory activity. Our methodology confirmed previously reported competitive and collaborative interactions among bacterial groups, which could be used either as marker of health status or as targets for modulation of the oral environment.

## Introduction

The oral cavity is inhabited by a large and complex microbial community (Aas et al., 2005) Structural, metabolic, and chemical interactions within the oral microbiome are fundamental for maintaining community homeostasis and a healthy microbiome (Hajishengallis et al., 2012). In this way, oral health entails microbiota in symbiosis with the host (Marsh, 2003). The unique structural characteristics of the teeth facilitate bacterial biofilm deposition over time, but excessive accumulation at the gingival margin triggers inflammation (Armitage, 2004). Further disruptions on the microbial homeostasis may promote the expression of community virulence factors from pathobionts (e.g., *Porphyromonas gingivalis, Tannerella forsythia, Treponema denticola*, and *Fusobacterium nucleatum*) (Socransky et al., 1998), resulting in dysbioses such as caries or periodontitis (Slomka et al., 2017). Recent studies have focused on exploring the community diversity in subjects with periodontal disease (Griffen et al., 2011), and in the structure shifts from health to periodontitis (Abusleme et al., 2013). However, taxonomic information may provide only partial comprehension of the function of each member within the community. Thus, principles such as substrate utilization must be considered when evaluating the impact of selected members of the oral microbiota on the host (Ramsey and Whiteley, 2009). This increased understanding of the metabolic activities of the oral microbiome may assist the successful management and personalized treatment of oral diseases in the future.

Cell response is a useful tool for characterizing performance variations resulting from environmental influences (Campbell et al., 2013). Metabolic similarity (Anderson et al., 2002) and functional diversity (Zhang et al., 2014) of dental biofilms have been studied, while additional techniques, such as genomics, have described cellular responses involved in periodontitis (Divaris et al., 2013). Decoding the metabolic associations between bacterial species is fundamental for the development of alternative therapies for oral disease. Nevertheless, the individual cell-specific substrate preferences of disease-associated, commensal, and/or health-associated bacteria have not been fully described. Phenomics entails the use of a phenotypic microarray (PM) to study changes in the cellular respiration through the detection of H^+^ ions, allowing for pathogen identification with either clinical or diagnostic potential (Bochner, 2009). In addition, analyzing metabolic variations between closely related species or between treated and control strains, is possible (Greetham, 2014).

In this study, we focused on investigating the impact of groups of specific molecules on the respiratory activity of selected members of the oral microbiome. Keystone pathogens such as *P. gingivalis* and *T. forsythia* are related to dysbiosis (Hajishengallis et al., 2012), while *Aggregatibacter actinomycetemcomitans* is found in aggressive periodontitis (Jiao et al., 2014) and as opportunistic pathogen (Stacy et al., 2016). Other bacteria, such as *Prevotella* spp. have increased prevalence on disease states (Hajishengallis and Lamont, 2012). Moreover, *Streptococcus mutans* is associated with peri-implantitis and caries (Maddi and Scannapieco, 2013). Other species such as *F. nucleatum* (Jiao et al., 2014), *Prevotella intermedia* (Maddi and Scannapieco, 2013), and *Streptococcus sobrinus* (Kilian et al., 2006) may act as putative pathogens. Health-associated bacterial community profiles are not well–understood in caries (Gross et al., 2010). Significant inverse correlations between caries and *S. gordonii*, but not *S. sanguinis* were reported (Gross et al., 2010), while other studies show increases in both species in caries vs. health (Peterson et al., 2013). On the contrary, *Streptococcus mitis, Streptococcus salivarius, Streptococcus oralis, Streptococcus sanguinis, Veillonella* sp., *Actinomyces* spp., and *Capnocytophaga* sp. have been reported in healthy individuals or negatively correlated with disease status (Socransky et al., 1998; Aas et al., 2005; Kilian et al., 2006; Maddi and Scannapieco, 2013; Loozen et al., 2014). Furthermore, *S. sanguinis* and *S. gordonii* were found to produce H_2_O_2_ and inhibit the growth of *S. mutans* and *A. actinomycetemcomitans* (Kreth et al., 2008; Sliepen et al., 2008). Commensal populations may prevent adhesion of pathogen species (Teughels et al., 2011). For instance, commensal biofilms containing *S. oralis, S. gordonii, S. mitis*, and *S. sanguinis* inhibited the planktonic and biofilm concentrations of *P. gingivalis* and *P. intermedia* (Herrero et al., 2016). Finally, species normally associated with dental health include *S. mitis, S. oralis*, and *S. sanguinis* (Peterson et al., 2013).

The fact that there is inconsistent definition about the functional roles of different species in health and disease was a rationale to perform this study. We selected the species described above to show a proof of concept of whether respiratory activity can be selectively promoted. We hypothesized that modulating bacterial respiratory metabolism using such functional compounds may enhance the fitness of bacteria performing potentially health-associated activities. We applied a multivariate statistical analysis to detect possible triggers common to several species. Nutrient availability may ultimately impact composition of a polymicrobial community (Hajishengallis and Lamont, 2016). Thus, if the requirements for certain metabolites are shared among the selected species, this may indicate that several species have the capacity to utilize the same metabolite. This approach for analyzing functional metabolic capacities may allow for explaining competitive and collaborative interactions among bacterial groups (Slomka et al., 2017). These individual requirements may certainly vary in live oral biofilms, but they can give us an indication on the metabolic strategies that bacteria with known functions (i.e., pathobionts) employ to persist in the biofilm. In addition, increased understanding of the metabolic capacities of members of the oral microbiome may be useful to highlight individual compounds. These can be used as markers for health status or for developing targets for modulation of the oral ecosystem.

## Materials and methods

### Bacterial cultures and collection

The following strains commonly present in the oral microbiome (Aas et al., 2005; Maddi and Scannapieco, 2013; Loozen et al., 2014) were obtained from the American Type Culture Collection (ATCC): *A*. *actinomycetemcomitans* (ATCC 43718), *F. nucleatum* (ATCC 10953), *P. gingivalis* (ATCC 33277), *P. intermedia* (ATCC 25611), *S. mutans* (ATCC 25175), *S. sobrinus* (ATCC 33478), *T. forsythia* (ATCC 43037), *Actinomyces naeslundii* (ATCC 51655), *Capnocytophaga sputigena* (ATCC 33612), *Streptococcus gordonii* (ATCC 49818), *Actinomyces viscosus* (ATCC 15987), and *S. mitis* (ATCC 49456). *Veillonella parvula* was obtained from the Leibniz Institute DSMZ-German Collection of Microorganisms and Cell Cultures (DSM 2007); *S. sanguinis* was acquired from the BCCM/LMG Bacteria Collection (LMG 14657). *S. salivarius* strain TOVE-R (Tanzer et al., 1985) was utilized. Bacterial strains were classified as either commensal (indigenous), potentially health-associated or disease-associated, as indicated in Table 1.

**Table 1:** Functional classification of the bacterial strains included in this study.

**Health-associated (Beneficial)**	**Indigenous (Commensal)**	**Disease-associated (Pathobionts)**
*Actinomyces viscosus* (ATCC 15987)	*Actinomyces naeslundii* (ATCC 51655)	*Aggregatibacter actinomycetemcomitans* (ATCC 43718)
*Streptococcus salivarius* TOVE-R	*Capnocytophaga sputigena* (ATCC 33612)	*Fusobacterium nucleatum* (ATCC 10953)
*Streptococcus mitis* (ATCC 49456)	*Streptococcus gordonii* (ATCC 49818)	*Porphyromonas gingivalis* (ATCC 33277)
*Streptococcus sanguinis* (LMG 14657)		*Prevotella intermedia* (ATCC 25611)
*Veillonella parvula* (DSM 2007)		*Streptococcus mutans* (ATCC 25175)
		*Streptococcus sobrinus* (ATCC 33478)
		*Tannerella forsythia* (ATCC 43037)

### Biolog PM assays

Biolog phenotype microarrays (PM, Biolog, Inc., Hayward, CA, USA) are a commercial high-throughput system for the analysis of microbial cellular phenotypes. The assay uses the reduction of a tetrazolium dye by NADH as a sensitive indicator of microbial respiration in response to the presence of individual nutrients or chemicals (Bochner et al., 2001). However, this methodology does not allow for estimating population size or bacterial abundance, because only a small portion of all compounds may be actively engaged in cell growth (Posch et al., 1997; Smith and Del Giorgio, 2003). PM plates 1–8, which contained 190 sole carbon sources, 95 sole nitrogen sources, 59 sole phosphate sources, 35 sole sulfur sources, 95 nutrient supplements, and 285 sole peptide-nitrogen sources were used in this analysis. All bacteria, excepting *T. forsythia*, were grown on blood agar plates (Blood Agar Base No 2, Oxoid, Hampshire, UK) supplemented with 5 μg/ml hemin (Sigma, Diegem, Belgium), 1 μg/ml menadione (Sigma, Diegem, Belgium), and 5% defibrinated horse blood (E&O Laboratories Limited, Bonnybridge, Scotland). *T. forsythia* was grown brain-heart infusion agar (Oxoid, Hampshire, UK) supplemented with 0.5% (w/v) of yeast extract (Oxoid, Hampshire, UK), 0.1% (w/v) of L-cysteine (Merck, Darmstadt, Germany), 5.0 mg/L of hemin (Sigma, Diegem, Belgium), 0.05 mg/L of menadione (Sigma, Diegem, Belgium), 0.001% (w/v) of N-acetylmuramic acid (Sigma, Diegem, Belgium), 5% Fetal Bovine Serum (Sigma, Diegem, Belgium), and 5% defibrinated horse blood (E&O Laboratories Limited, Bonnybridge, Scotland). *A. actinomycetemcomitans* (Aa), *S. gordonii* (Sgord), *S. mitis* (Smit), *S. mutans* (Smut), *S. salivarius* (Ssal), *S. sanguinis* (Ssang), and *S. sobrinus* (Ssob) were incubated at 37°C in a 5% CO_2_ environment. *A. naeslundii* (Anaes), *A. viscosus* (Avisc), *C. sputigena* (Csput), *F. nucleatum* (Fn), *P. gingivalis* (Pg), *P. intermedia* (Pi), *T. forsythia* (Tf), and *V. parvula* (Vp) were incubated at 37°C under anaerobic conditions (10% CO_2_, 10% H_2_, 80% N_2_). Anaerobic incubation was performed to recreate the conditions in the periodontal pockets (Marsh et al., 2015). PM assays were performed following the manufacturer's instructions using Biolog Redox Dye Mix D. Metabolic activity was estimated based on the optical density (OD), which was measured after 0, 24, and 48 h of incubation under anaerobic conditions at 37°C at 492 nm using a microplate reader (Multiskan Ascent, Thermo Fisher Scientific Oy, Vantaa, Finland). Incubations were performed in triplicate, under anaerobic conditions (10% CO_2_, 10% H_2_, 80% N_2_), because all bacterial strains were facultative or anaerobic.

### Multivariate statistical analysis

All the metabolites were categorized according to the respiratory activity triggered on each bacterial species, regarding the control (no nutrient added). Mean OD was calculated for each metabolite, based on the OD readings from the 15 bacterial species. If the OD reading on a species was above the 0.975 quantile, the metabolite was considered to generate high respiratory activity (H-RA) in that strain. Metabolites whose OD value was below the 0.025 quantile were considered to trigger low respiratory activity (L-RA). If the OD was between the 0.025 and 0.975 quantiles, it was considered not significantly different from the control (NS); control OD readings were included within this category (Hernandez-Sanabria et al., 2010). The frequency of the activity triggered by each metabolite within a group of bacteria (commensal, disease-associated, or health-associated) was compared using a Chi-square analysis. In this way, 3 × 2 contingency tables of cross-classification containing the respiratory activity (H, L, or NS) were produced for each of the bacterial groups. When the count of any of the cells was below 5, Fisher Exact test (R Core Team, 2000) was used to calculate the table probability (Hernandez-Sanabria et al., 2013), and activity-associated metabolites were highlighted.

Multiple Correspondence Analysis (MCA) was applied to detect similarities in the respiratory activity of the 15 species. The coordinates best representing bacterial species on two dimensions were retained to create clusters of bacteria with similarities in the use of the PM microarray metabolites. Thus, Hierarchical Clustering of Principal Components (HCPC) was performed, followed by construction of trees using the Ward's method (double factor hierarchical clustering) (Husson et al., 2010). Further, the Calinski-Harabasz (CH) index on k-means clustering was used to evaluate the cluster validity based on the average between- and within- cluster sum of squares, using the vegan package in R (Oksanen et al., 2013). The package NbClust in R was employed to calculate the CH, Silhouette, and Dunn indices (Charrad et al., 2014). Scores were visualized with the package factoextra (Kassambara and Mundt, 2016). Multiple Factor Analysis (MFA) was employed to discriminate particular metabolites triggering respiratory activity (H-RA, L-RA, NS) on each bacterial group at both time points. The function MFA from the FactoMineR package (Lê et al., 2008) was performed in R and the square cosine values (*cos*^2^) extracted. Results were plotted in a Principal Coordinate Analysis (PCoA) (Oksanen et al., 2013) and visualized (Wickham, 2016).

### Flow cytometry analysis

Further proof-of-principle validation was performed in selected species grown in complex medium, to determine whether activity can nevertheless be increased by specific compounds. Cell number and membrane integrity were used as indicators of activity (De Roy et al., 2012). Culturing of *F. nucleatum, A. actinomycetemcomitans*, and *S. salivarius* was in modified brain-heart infusion (Alvarez et al., 2013), using the Hungate tube method and maintaining the media in anaerobic conditions (10% CO_2_, 10% H_2_, 80% N_2_). Optical density of the culture used for inoculation was measured after 18 h. Growth analyses on single nutrient sources were performed in 8.0 ml of medium, supplemented with 1% v/v of either L-Asparagine, L-Aspartic Acid, or D-Sorbitol (Sigma, Diegem, Belgium), all at 25mM. Triplicate tubes were inoculated with 100 μl of a 18-h culture diluted 10 × and further incubated anaerobically at 37°C. Flow cytometry was performed at 0, 24, and 48 h. SYBR Green I (SG, Invitrogen, Merelbeke, Belgium) was used alone or in combination with Propidium Iodide (Live/dead BacLight Bacterial Viability kit, Invitrogen, Merelbeke, Belgium), to quantify the total number of bacteria and to differentiate between cells with intact and damaged cytoplasmic membranes, respectively (Berney et al., 2007). Stock staining solutions were prepared as described elsewhere (Van Nevel et al., 2013). Under anaerobic conditions, samples were placed in 96-well plates, diluted 100 × with PBS (pH 7.0) and stained with 2 μl of staining solution and 2 μl of 500 mM Na_2_EDTA (pH 8.0) for outer membrane permeabilization. Prior to flow cytometry analysis, the stained samples were incubated for 13 min in the dark at 37°C. Cell count analysis was performed using an Accuri C6 flow cytometer (BD Biosciences, Erembodegem, Belgium) equipped with an autoloader, at a flow rate of 66 μl min^−1^ in 25 μl (Van Nevel et al., 2013). Processing of the flow cytometry results was performed with the Summit v4.3 software. Differences in total and intact number of bacterial cells supplemented with different metabolites were compared using a mixed model with time as a repeated measure, in SAS version 9.3 (Sas, 2012). Statistical significance was assumed at *P* < 0.05.

## Results

### Respiratory activity in response to diverse metabolic triggers was different for each bacterial group

The results of the tetrazolium dye indicator assay were used as estimates of the respiratory activity. Changes regarding the control were classified into High respiratory activity (H-RA), Low respiratory activity (L-RA), or Not Significantly different (NS) from the control using a Chi-square procedure. In this way, 3 × 2 contingency tables of cross-classification containing the respiratory activity (H, L, or NS) were produced for each of the bacterial groups (health-associated, disease-associated, or commensals). The objective of this analysis was to detect common metabolites that promote similar respiratory activity on an entire bacterial group.

**Figure 1:**
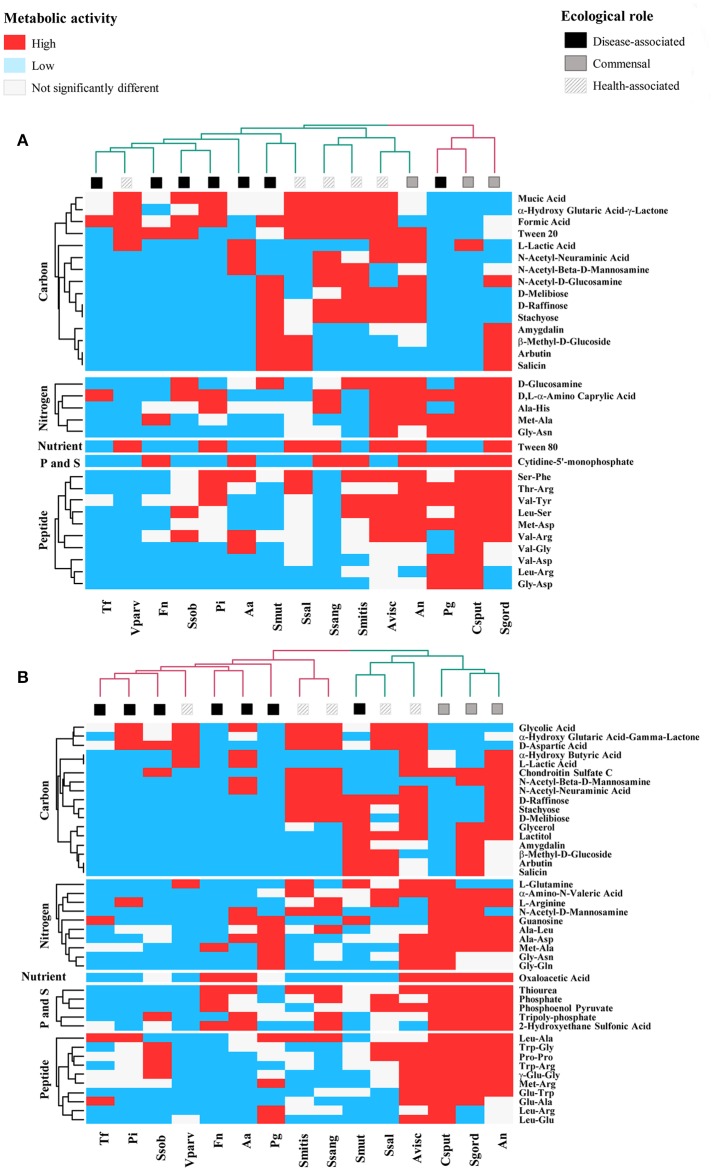
Specific Biolog substrates correlated with high respiratory activity at **(A)** 24 h and **(B)** 48 h, in health-associated (*n* = 5), commensal (*n* = 3), and disease-associated bacteria (*n* = 7). Substrates in red significantly increased the respiratory activity in regards to the control (*P* < 0.05). Blue squares indicate metabolites that significantly decrease the respiratory activity and white squares are for compounds that had no significant effect on the selected bacteria. Table 2 includes the complete description of the metabolites, the significant differences in their effects on the three groups of bacteria and *P*-values.

Thus, Val-Ala increased the respiratory activity in five of the disease-associated species but the effect was not significantly different from the control for *F. nucleatum* and in *P. gingivalis*. Additional 17 compounds tended to decrease the respiratory activity of disease-associated at 24 h (*n* = 7, *P* < 0.10, Figure 1A and Table 2A). From these, 11 were carbon sources, one nitrogen and one nutrient source, and four peptides. The respiratory activity of all the commensal species (*n* = 3), was increased with sources of nitrogen and peptides. Only carbon sources triggered high respiratory activity (*P* < 0.0001) in all the health-associated (*n* = 5). At 48 h, 2-aminoethanol increased the respiratory activity of 5 disease-associated bacteria, but not in *P. gingivalis* and *A. actinomycetemcomitans* (Figure 1B). Similarly, glucuronamide stimulated respiration in all disease-associated except for *F. nucleatum* and *P. gingivalis*. A total of 14 carbon, four nitrogen sources, and three peptides tended to decrease the respiratory activity of all disease-associated, but not significantly (Table 2B). For the commensals, 20 compounds promoted their respiratory activity, mainly peptides (*P* < 0.0001). Three sources of carbon increased the respiratory activity of all health-associated species (*P* < 0.0001). Three molecules triggered activity at both 24 and 48 h in commensals, whereas α-hydroxyglutaric acid γ-lactone had similar impact in health-associated. None of the evaluated compounds significantly increased activity in disease-associated at both time points (Figure 1B). Our results indicated that the metabolite requirements of disease-associated bacteria are dissimilar, suggesting that collective modulation of their respiratory activity may be challenging. However, one molecule may consistently support the respiratory activity of all species considered health-associated.

**Table 2:** Metabolites associated with respiratory activity changes in oral bacteria at 24 and 48 h (*n* = 15).

**Disease-associated (*****n*** = **7)**				**Commensal (*****n*** = **3)**				**Health-associated (*****n*** = **5)**			
**Metabolite**		**Frequency (%)**		**Metabolite**		**Frequency (%)**		**Metabolite**		**Frequency (%)**	
		**H-RA**	**L-RA**			**H-RA**	**L-RA**			**H-RA**	**L-RA**
**(A)**											
C	β-Methyl-D-Glucoside	14.3 (Smut)	85.7^*^	N	Ala-His	100.0	0.0^**^	C	α-Hydroxy Glutaric Acid-γ-Lactone	100.0	0.0^**^
C	D-Melibiose	14.3 (Smut)	85.7^*^	N	D, L-α-Amino Caprylic Acid	100.0	0.0^**^	C	Formic Acid	100.0	0.0^**^
C	L-Lactic Acid	14.3 (Aa)	85.7^*^	N	D-Glucosamine	100.0	0.0^**^	C	Mucic Acid	100.0	0.0^**^
C	N-Acetyl-β-D-Mannosamine	14.3 (Aa)	85.7^*^	N	Met-Ala	100.0	0.0^**^	C	Tween 20	100.0	0.0^**^
C	N-Acetyl-D-Glucosamine	14.3 (Smut)	85.7^*^	PS	Cytidine-5'-monophosphate	100.0	0.0^**^				
C	Amygdalin	14.3 (Smut)	85.7^*^	PEP	γ-Glu-Gly	100.0	0.0^**^				
C	Arbutin	14.3 (Smut)	85.7^*^	PEP	Met-Arg	100.0	0.0^**^				
C	D-Raffinose	14.3 (Smut)	85.7^*^	PEP	Ser-Met	100.0	0.0^**^				
C	N-Acetyl-Neuraminic Acid	14.3 (Aa)	85.7^*^	PEP	Thr-Ala	100.0	0.0^**^				
C	Salicin	14.3 (Smut)	85.7^*^	PEP	Tyr-Trp	100.0	0.0^**^				
C	Stachyose	14.3 (Smut)	85.7^*^	PEP	Val-Ile	100.0	0.0^**^				
N	Gly-Asn	14.3 (Pg)	85.7^*^								
NU	Tween 80	14.3 (Pi)	85.7^*^								
PEP	Leu-Arg	14.3 (Pg)	85.7^*^								
PEP	Val-Arg	14.3 (Pg)	85.7^*^								
PEP	Val-Asn	14.3 (Aa)	85.7^*^								
PEP	Gly-Asn	14.3 (Pg)	85.7^*^								
**(B)**											
C	α-Hydroxy Butyric Acid	14.3 (Aa)	85.7^*^	C	Chondroitin Sulfate C	100.0	0.0^**^	C	α-Hydroxy Glutaric Acid-γ-Lactone	100.0	0.0^**^
C	β-Methyl-D-Glucoside	14.3 (Smut)	85.7^*^	N	Ala-Asp	100.0	0.0^**^	C	D-Aspartic Acid	100.0	0.0^**^
C	D-Melibiose	14.3 (Smut)	85.7^*^	N	Ala-Leu	100.0	0.0^**^	C	Glycolic Acid	100.0	0.0^**^
C	Glycerol	14.3 (Smut)	85.7^*^	N	α-Amino-N-Valeric Acid	100.0	0.0^**^				
C	L-Lactic Acid	14.3 (Aa)	85.7^*^	N	Guanosine	100.0	0.0^**^				
C	N-Acetyl-β-D-Mannosamine	14.3 (Aa)	85.7^*^	N	L-Arginine	100.0	0.0^**^				
C	Amygdalin	14.3 (Smut)	85.7^*^	N	Met-Ala	100.0	0.0^**^				
C	Arbutin	14.3 (Smut)	85.7^*^	PS	2-Hydroxyethane Sulfonic Acid	100.0	0.0^**^				
C	Chondroitin Sulfate C	14.3 (Smut)	85.7^*^	PS	Phosphate	100.0	0.0^**^				
C	D-Raffinose	14.3 (Ssob)	85.7^*^	PS	Phosphoenol Pyruvate	100.0	0.0^**^				
C	Lactitol	14.3 (Smut)	85.7^*^	PS	Thiourea	100.0	0.0^**^				
C	N-Acetyl-Neuraminic Acid	14.3 (Aa)	85.7^*^	PS	Tripoly-phosphate	100.0	0.0^**^				
C	Salicin	14.3 (Smut)	85.7^*^	NU	Oxaloacetic Acid	100.0	0.0^**^				
C	Stachyose	14.3 (Smut)	85.7^*^	PEP	Glu-Trp	100.0	0.0^**^				
N	Gly-Asn	14.3 (Pg)	85.7^*^	PEP	Leu-Ala	100.0	0.0^**^				
N	Guanine	14.3 (Pg)	85.7^*^	PEP	γ-Glu-Gly	100.0	0.0^**^				
N	L-Glutamine	14.3 (Smut)	85.7^*^	PEP	Met-Arg	100.0	0.0^**^				
N	N-Acetyl-D-Mannosamine	14.3 (Aa)	85.7^*^	PEP	Pro-Pro	100.0	0.0^**^				
PEP	Leu-Arg	14.3 (Pg)	85.7^*^	PEP	Trp-Arg	100.0	0.0^**^				
PEP	Leu-Glu	14.3 (Pg)	85.7^*^	PEP	Trp-Gly	100.0	0.0^**^				
PEP	Glu-Ala	14.3 (Tf)	85.7^*^								

H-RA, high respiratory activity; L-RA, low respiratory activity;

* P < 0.10;

** *P < 0.05. C, carbon source; N, nitrogen source; NU, nutrient source; PS, phosphorus and/or sulfur source; PEP, peptide source*.

^*^In parenthesis, species that increased their respiratory activity with the indicated metabolite: Smut, Streptococcus mutans; Aa, Aggregatibacter actinomycetemcomitans; Ssob, Streptococcus sobrinus; Pi, Prevotella intermedia; Pg, Porphyromonas gingivalis; Tf, Tannerella forsythia.

### Functional similarities among bacterial groups shift over time

**Figure 2:**
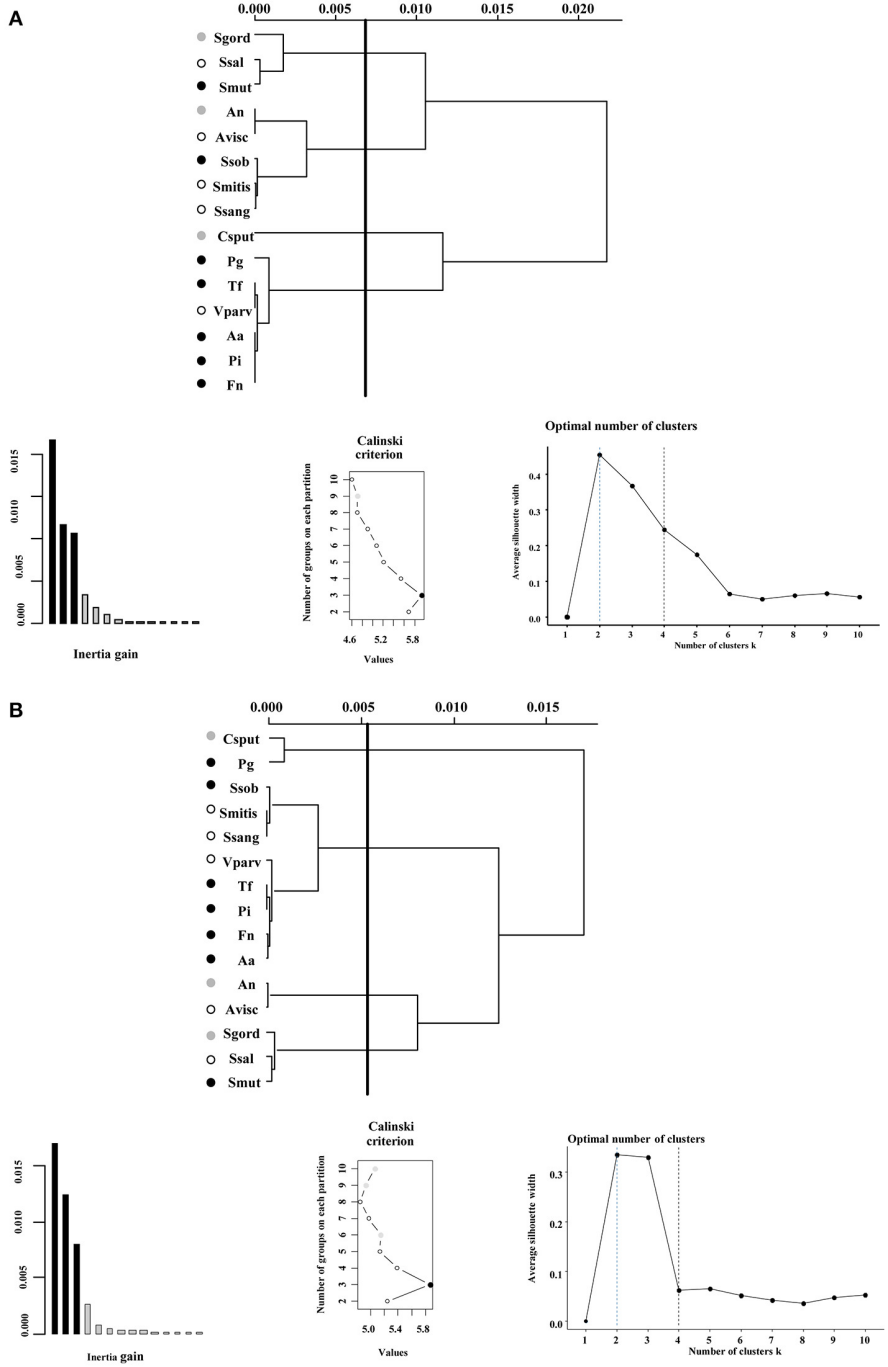
Ward's method of hierarchical unsupervised clustering based on the respiratory activity triggered by substrates contained in the Biolog Phenotype microarray at **(A)** 24 h and **(B)** 48 h. The first 2 principal components of the multiple correspondence analysis were retained for cluster analysis. The *x*-axis of dendrogram represents the linkage distance measured by the Euclidean distance squared. The *y*-axis indicates the bacterial species included on each cluster. Brackets outline the clusters statistically significant at the 0.05% level and a vertical line indicates this cut-off linkage distance. The groups discussed in the text are colored as follows: health-associated species in white, commensals in gray, and disease-associated bacteria in black circles. Validation metrics of the clustering performance are included in the lower panel. On the left, the inertia explained by each of the clusters; on the center, the Calinski-Harabasz (CH) score indicating the optimal number of clusters; and on the right, the Silhouette scores. CH scores at different number of clusters are described in Supplementary Table 1.

**Figure 3:**
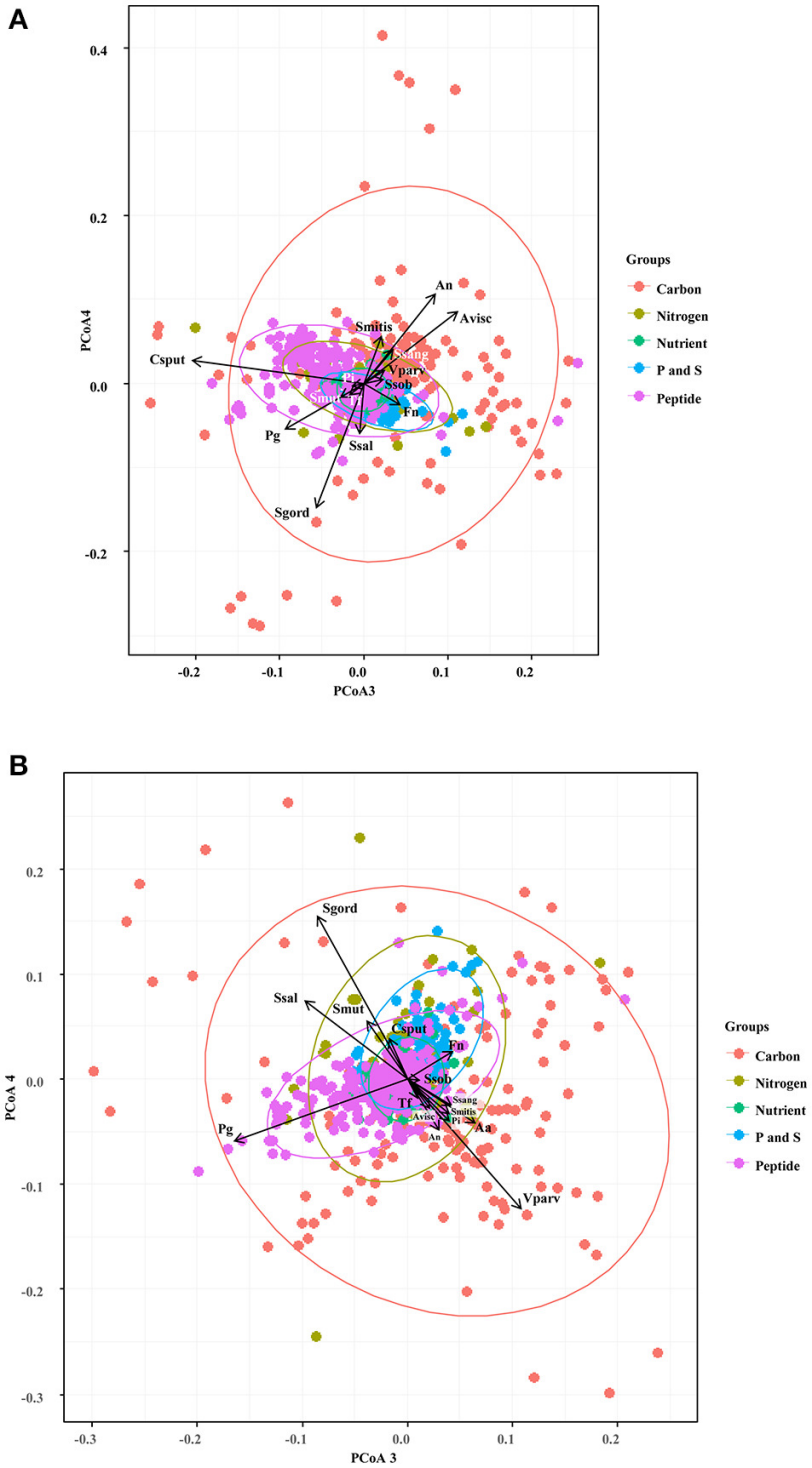
Specific groups of metabolites influenced respiratory activity of each bacterial community. Principal Coordinate Analysis assisted to explain the grouping trends in the cluster analysis, and highlighted similarities in the respiratory activity among the selected oral bacteria at 24 h **(A)** and 48 h **(B)**. Aa, *Aggregatibacter actinomycetemcomitans* (ATCC 43718); Fn, *Fusobacterium nucleatum* (ATCC 10953); Pg, *Porphyromonas gingivalis* (ATCC 33277); Pi, *Prevotella intermedia* (ATCC 25611); Sm, *Streptococcus mutans* (ATCC 25175); Ssob, *Streptococcus sobrinus* (ATCC 33478); Tf, *Tannerella forsythia* (ATCC 43037); An, *Actinomyces naeslundii* (ATCC 51655); Csput, *Capnocytophaga sputigena* (ATCC 33612); Sgord, *Streptococcus gordonii* (ATCC 49818); Avisc, *Actinomyces viscosus* (ATCC 15987); Smitis, *Streptococcus mitis* (ATCC 49456); Vparv, *Veillonella parvula* (DSM 2007); Ssang, *Streptococcus sanguinis* (LMG 14657), and Ssal, *Streptococcus salivarius* strain TOVE-R.

We first focused in detecting the metabolites that collectively increased the respiratory activity on each of the bacterial groups tested, using the frequency analysis. Then, MCA was employed to determine whether bacteria could be grouped by overall metabolic capacities and requirements, regardless of their initial classification (health-associated, disease-associated or commensal). The loadings of the first five components of the MCA were retained and clustered using the k-means hierarchical clustering (Supplementary Table 1). At 24 h, commensals and health-associated were grouped together while a second cluster included five disease-associated, one commensal, and one health-associated (Figure 2A). *C. sputigena* showed functional similarities with *P. gingivalis* at 48 h and their activity was significantly different from the bacteria grouped in the other clusters (*P* < 0.05, Figure 2B). *S. mitis, S. sanguinis*, and *V. parvula* were clustered with disease-associated, whereas *A. naeslundii, A. viscosus, S. gordonii*, and *S. salivarius* remained together over time. This clustering analysis suggested that there may be overall metabolic similarities among bacterial species. Following, MFA was applied to explain why bacteria considered as commensal or health-associated were grouped with disease-associated. Based on the respiratory activity triggered by all the screened metabolites, health-associated were included in dimensions 3 and 4 at 24 h (Figure 3A). Commensal bacteria were also included in Dim 3, where the highest scores from the metabolic variables corresponded to the carbon and nitrogen sources (Supplementary Table 2A). This result indicates that carbon and nitrogen sources promote respiratory activity in both health-associated and commensals. At 48 h, health-associated bacteria were spread in Dimension 4 and 5, while disease-associated were in Dimension 3 and commensals in Dim. 2. (Figure 3B). The location of health-associated bacteria in the plot, regarding disease-associated, indicated that the differences in the metabolic activity among both groups were triggered by phosphorus and sulfur (Supplementary Table 2B). These findings confirmed that metabolic requirements in health-associated species fluctuate over time but they are shared among the members of a specific bacterial community.

### Potential competitive interactions influence the fitness of the oral ecosystem members

MFA was additionally used to discriminate metabolites that exclusively triggered respiratory activity in a single group of bacteria, and to uncover correlations among these metabolites. The square correlation ratios (*cos*^2^) measure the degree of association between variables and any given dimension of the factor analysis. Thus, the *cos*^2^ between the coordinates of the bacterial species and the metabolites revealed that carbon and nitrogen sources were the main metabolic triggers for health-associated bacteria at 24 h 1 (*cos*^2^ > 0.8, Supplementary Table 2A). Ala-Gly, urea, D, L-α-Amino-N-Butyric Acid, L-Phenylalanine, nitrate, L-Cysteine, α-Amino-N-Valeric Acid, L-Citrulline, L-Asparagine, D-Lysine, D-Glucosamine, L-Lysine, and ammonia had the highest contributions to dimensions 3 and 4 of the MFA, which both described health-associated bacteria. This suggested that the above were the metabolites that triggered high respiratory activity only in all the health-associated (Supplementary Table 2). After 48 h, carbon and P and S sources were the metabolites that contributed to discriminate between disease-associated and health-associated bacteria. L-Alanyl-Glycine, L-Methionine, Ala-Gly, γ-Hydroxy Butyric Acid, and D-Glucose-1-Phosphate, were described in Dimension 3 with disease-associated, while Glycyl-L-Proline, Glycyl-L-Aspartic Acid, Glycyl-L-Glutamic Acid, Gly-Asn, L-Leucine, gelatine, and L-Alanyl-Glycine, 2-Hydroxyethane Sulfonic Acid, and L-Cysteine Sulfinic Acid, were included in Dimension 4, with health-associated species (Supplementary Table 2B, *P* < 0.05). L-Valine, L-Isoleucine, and O-Phospho-D-Tyrosine were positively associated with both health-associated and disease-associated, indicating that both groups increase their respiratory activity with these compounds, suggesting potential competitive interactions. L-Glutamine and N-Acetyl-L-Cysteine were negatively associated with disease-associated and Adenosine-3',5'-cyclic monophosphate, and Taurine were partially associated with health-associated, because their *cos*^2^ values were high on either dimension 4 or 5 (Supplementary Table 2B). These results explain why collective modulation of respiratory activity may result challenging and thus, alternative strategies may need to be developed.

### Metabolic activity is not solely attributable to increased cell number

Flow cytometry was used to validate whether variations in the respiratory activity were a result of increased cell number. Bacterial cells were quantified following supplementation with compounds that either promoted respiratory activity (L-Asparagine, in health-associated) or that were not found to have effect on the respiratory activity of neither group (D-Sorbitol and L-Aspartic Acid). SYBR Green I (SG) allows for quantification of the total cell number irrespective of viability or cultivability (Zipper et al., 2004), while Propidium iodide (PI) is appropriate for investigating oxidative damage to cells. We used them in combination to differentiate between intact and damaged cells. Significant differences in total cell number in comparison with the control were identified only when L-Aspartic Acid was supplemented (*P* < 0.05, Table 3). PI allowed for determination of the impact that diverse metabolites may have on the membrane integrity. L-Asparagine and L-Aspartic Acid decreased the number of intact cells in *F. nucleatum* and *S. salivarius* at 48 h. However, no significant difference in cell numbers of *F. nucleatum* was detected with either stain at 24 h. Intact cells of *S. salivarius* were less when sources of amino acids were added to the medium, while the opposite effect was observed in *A. actinomycetemcomitans* (*P* < 0.05). These results indicate that the metabolic activity detected in the Biolog plates may not be a direct result of the number of bacterial cells. In addition, damage to the cell membrane may not influence overall respiratory activity.

**Table 3:** Total **(A)** and Intact **(B)** cell number of selected oral inhabitants at 24 and 48 h.

**Time point**	**Species**	**Treatment**	**Mean cell count (Log units)**	**SEM**	***P*-value**
**(A)**					
24 h	Aa	CTR	8.56^a^	0.04	0.0005
		LAA	8.24^b^		
		LAS	8.56^a^		
		SOR	8.57^a^		
	Fn	CTR	8.14	0.23	0.22
		LAA	7.72		
		LAS	7.54		
		SOR	8.17		
	Ss	CTR	8.36^a^	0.05	<0.0001
		LAA	7.80^b^		
		LAS	8.28^a^		
		SOR	8.37^a^		
48 h	Aa	CTR	8.79^a^	0.03	<0.0001
		LAA	8.39^b^		
		LAS	8.71^a^		
		SOR	8.78^a^		
	Fn	CTR	8.54^a^	0.07	0.01
		LAA	8.16^b^		
		LAS	8.30^a, b^		
		SOR	8.55^a^		
	Ss	CTR	8.41^a^	0.04	<0.0001
		LAA	7.65^b^		
		LAS	8.38^a^		
		SOR	8.48^a^		
**(B)**					
24 h	Aa	CTR	7.31^b^	0.06	0.0003
		LAA	7.84^a^		
		LAS	7.70^a^		
		SOR	7.20^b^		
	Fn	CTR	7.97	0.26	0.13
		LAA	7.32		
		LAS	7.23		
		SOR	8.01		
	Ss	CTR	7.17^a^	0.13	0.002
		LAA	6.31^b^		
		LAS	7.29^a^		
		SOR	7.13^a^		
48 h	Aa	CTR	8.35^a^	0.13	0.004
		LAA	7.59^b^		
		LAS	8.39^a^		
		SOR	8.56^a^		
	Fn	CTR	8.25^a, b^	0.09	0.06
		LAA	8.02^b^		
		LAS	8.05^b^		
		SOR	8.37^a^		
	Ss	CTR	7.35^a^	0.06	<0.0001
		LAA	6.15^b^		
		LAS	7.33^a^		
		SOR	7.40^a^		

*CTR, control; LAA, L-Aspartic Acid; LAS, L-Asparagine; SOR, D-Sorbitol. Aa, Aggregatibacter actinomycetemcomitans; Fn, Fusobacterium nucleatum; Ss, Streptococcus salivarius. Means with same letter are not significantly different across treatments*.

## Discussion

As suggested by numerous studies, the oral ecosystem displays synergic activity (Kolenbrander et al., 2010; Tonetti et al., 2011). In the current study, we determined potential substrates that can be used by a wide range of oral bacteria. Multivariate analysis was used to support our hypothesis that specific molecules can selectively stimulate health-associated bacteria. We aimed at explaining whether the metabolic activities could clarify why bacteria considered commensal are frequently accompanying disease-associated bacteria, while some other times they are linked to oral health. Thus, we showed how different bacteria have different responses to such molecules.

Although the respiratory activity was assessed individually, our results may potentially serve as indicators of functional triggers shared by different populations. The commercially available assays of cell-specific metabolic activity in bacteria provide a direct measurement only of the responsive bacterial cells performing respiration. Thus, cell-specific respiration rates may not represent total bacteria abundance (Smith and Del Giorgio, 2003). In fact, a small portion of this abundance may potentially account for the overall activity detected (Posch et al., 1997), which was suggested by the flow cytometry results.

Few substrates increased the respiratory activity of all disease-associated bacteria, indicating that each species may follow different metabolic pathways to generate dysbiosis. Although disease-associated bacteria clustered all together at 24 h, *P. gingivalis* grouped separately after 48 h. *V. parvula* was the only health-associated bacteria in the same cluster of the disease-associated, suggesting metabolic similarities, regardless time. Previous reports revealed that *A. actinomycetemcomitans* grows in the presence of *Veillonella* sp. and/or *F. nucleatum* (Periasamy and Kolenbrander, 2009), while *F. nucleatum* enables growth of *P. gingivalis* by scavenging O_2_ (Diaz et al., 2004). Moreover, *S. gordonii, Actinomyces* sp., *Veillonella* sp. and *A. actinomycetemcomitans* are associated with *P. gingivalis*, because *P. gingivalis* provides pH support during supragingival colonization (Periasamy and Kolenbrander, 2009). Additionally, our analysis revealed that streptococci clustered in two groups, independently from genetic relatedness (e.g., *S. gordonii* and *S. sanguinis* are closely related, while *S. mutans* and *S. sobrinus* belong to the same subgroup of streptococci; Xu et al., 2014). Previous reports proposed that the distinction between dental health and disease may be independent of the overall abundance of *Streptococcus* spp. but rather linked to relative rates of acid production (Peterson et al., 2013). In this way, the fitness of periodontal pathobionts to colonize dental plaque may be shaped by both synergistic and competitive interactions with other members of the oral ecosystem (Jakubovics, 2010). Even though we separately examined the respiratory activities of 15 members of the oral plaque, the results of our clustering analyses presented associations validated in other systems (Kumar and Mason, 2015).

The relevance of carbon and nitrogen sources for the metabolism of health-associated bacteria, inferred in our analysis, (Figure 3) may aid to explain competitive interactions detected when bacteria are grown in biofilms (Jakubovics and Palmer, 2013). For instance, it has been reported that *F. nucleatum* (Bolstad et al., 1996), *P. gingivalis* (Mazumdar et al., 2009), and *T. forsythia* (Cross et al., 2014) prefer amino acids, while carbon is utilized by *A. actinomycetemcomitans* (Brown and Whiteley, 2007). Peptides are necessary for *P. gingivalis, P. intermedia* (Takahashi, 2005) and are the second most important energy source for *F. nucleatum* (Kapatral et al., 2002), while valine, leucine and isoleucine are required for *P. intermedia* and *S. sanguinis* (Takahashi, 2005). Our analysis confirmed that L-Leucine increased the respiratory activity of health-associated bacteria (Supplementary Table 2B). *P. gingivalis* and *S. sanguinis* have evolved to employ “energetically cheaper” amino acids for protein biosynthesis, as opposed to *T. forsythia* and *P. intermedia* (Cross et al., 2014). Consequently, using peptide uptake transport systems may influence the respiratory activity of the health-associated species, and possibly modulate *in vivo* competitive interactions, as observed with other disease-associated bacteria (Hajishengallis and Lamont, 2016).

The nitrogen released or transferred from deamination or transamination of amino acids results in carbon skeletons that can be used via glucogenic or ketogenic catabolic pathways. In this way, amino acids potentially supported the respiratory activity of health-associated bacteria. Indeed *Bacilli* (*Streptococcus* spp.), *Fusobacteria, Bacteroidetes* (*Prevotella, Tannerella* spp. and *Porphyromonas* spp.), *Actinobacteria* (*Actinomyces* spp.), Gammaproteobacteria (*Aggregatibacter* spp.), and Negativicutes (*Veillonella* spp.) are capable of synthesizing methionine from homoserine (Ferla and Patrick, 2014). In this way, the increase of respiratory activity when health-associated bacteria were exposed to amino acids, may explain competitive interactions observed in oral biofilms *in vivo* (Slomka et al., 2017). Further, we observed that L-glutamine, a precursor of leucine and glutamic acid, increased the respiratory activity of *S. mutans* after 48 h. The ability of this species to survive long-term low pH may be associated with the biosynthesis of branched chain amino acids from L-glutamine (Krzyściak et al., 2014). Glucuronamide and ethanolamine (2-aminoethanol) can be used for cellular membrane synthesis (Hendrickson et al., 2014) and as sources of carbon for pathogens (Chiok et al., 2013). The increased respiratory activity of *F. nucleatum and P. gingivalis* at 48 h when glucuronamide was supplied, suggested that their reported coaggregation interactions (Kolenbrander et al., 2010), may be based not only on localization but on the utilization of common resources and similar metabolic activities.

The boost in the respiratory activity triggered by α-hydroxyglutaric acid-γ-lactone in health-associated may be explained by its role in the pentose phosphate pathway, a major source of reducing equivalents for biosynthesis reactions. The complete genome of *S. salivarius* JIM8777 in the NCBI database indicated the presence of an enol-lactone hydrolase, confirming its potential ability of using lactones. Moreover, lactonizing enzymes perform “house-cleaning” functions and prevent the accumulation of toxic, non-degradable metabolites (Galperin et al., 2006). The ability of health-associated bacteria for using lactones as a potential source of energy (Mochizuki, 2001), and for membrane functions suggests that health-associated may be more efficient at sensing and communicating variations in the surrounding environment (Kumar and Mason, 2015). Later biofilm formation could be triggered by and include pathways utilizing other molecules pinpointed in our analysis. However, phenotypic variation among strains and genetic relatedness may govern how commensal relationships are ultimately transformed into pathogenic (Kilian et al., 2006; Xu et al., 2014).

Our results suggest that disease-associated bacteria inhabit distinct ecological niches defined not only by physical location and resource utilization (Socransky et al., 1998), but also by their level of metabolic activity (Maurice et al., 2013). Although the increase in respiratory activity triggered by some metabolites may be strain-dependent, we inferred metabolites that might explain the potential mechanisms that oral bacteria employ to adjust their habitat. In this way, it is possible that specific molecules can selectively stimulate health-associated bacteria, as indicated in previous work (Slomka et al., 2017). Combinations of these species in biofilms are likely to buffer the effects of key metabolic substrates identified. For this reason, future efforts to elucidate the roles of specific molecules, using whole-genome sequencing will be necessary for a complete understanding of the observed shifts. Our study provides a framework to potentially identify how the variations in the metabolic activity of individual key players may impact the later biofilm formation. These results may aid to elucidate the functional diversity and metabolic potential of the growing and metabolically active individual members of the oral biofilm. This description of potential functional targets supplies a preliminary outlook that can assist to further develop strategies to manage oral dysbiosis, and improve overall oral health.

## Author contributions

Conceived and designed the experiments: VS, WT, and NB. Performed the experiments: VS. Methodology development: EH-S, VS, ERH, WT, and NB. Data mining, statistical analysis, results interpretation, figure, and table preparation: EH-S. Writing the code for frequency analysis in R: FK with the guidance of EH-S. Funding acquisition: NB, WT, and LZ. Contributed reagents/materials/analysis tools: NB, WT. Wrote the paper: EH-S. Reviewed the manuscript: EH-S, VS, ERH, FK, WT, LZ, and NB.

### Conflict of interest statement

The authors declare that the research was conducted in the absence of any commercial or financial relationships that could be construed as a potential conflict of interest.
