# Supplementary material for: In vitro Increased Respiratory Activity of Selected Oral Bacteria May Explain Competitive and Collaborative Interactions in the Oral Microbiome
Source: Front Cell Infect Microbiol. 2017 Jun 7;7:235. doi: 10.3389/fcimb.2017.00235 (PMC5461333; doi:10.3389/fcimb.2017.00235)
Supplement: Supplementary file 1 [file Table1.DOCX]

Supplementary Material

Increased respiratory activity of individual members may explain further interactions in the oral microbiome

**Emma Hernandez-Sanabria^1,4^, Vera Slomka^2,4^, Esteban Rodriguez Herrero^2^, Frederiek-Maarten Kerckhof^1^, Lynette Zaidel^3^, Wim Teughels^2^, and Nico Boon^1*^**

**^1^** Center for Microbial Ecology and Technology (CMET), Ghent University. Coupure Links 653, B-9000 Gent, Belgium

**^2^** Department of Oral Health Sciences, KU Leuven. Kapucijnenvoer 33, B-3000 Leuven, Belgium

**^3^** Colgate-Palmolive Technology Center. 909 River Rd, Piscataway, NJ, 08854, USA

*** Correspondence:**

Nico Boon, Ghent University, Faculty of Bioscience Engineering, Center for Microbial Ecology and Technology (CMET), Coupure Links 653, B-9000 Gent, Belgium, phone: +32 (0)9 264 59 76, fax: +32 (0)9 264 62 48.

E-mail: [Nico.Boon@UGent.be](mailto:Nico.Boon@UGent.be)

**^4^** These authors contributed equally to this work

Supplementary Table 1. Performance validation indexes for k-means clustering of respiratory activity of selected members of the oral microbiome. The high value of the index at three clusters indicates that three is the optimal number of clusters that can be applied to comprise bacterial species with potentially similar respiratory activities.

| Time point | Number of clusters | Index | | |
| --- | --- | --- | --- | --- |
|  |  | Calinski-Harabasz | Silhouette | Dunn |
| 24 h | 2 | 5.6837 | 0.5215 | 0.9610 |
|  | 3 | **5.9294** | **0.5016** | **0.9764** |
|  | 4 | 5.5361 | 0.4446 | 0.9083 |
|  | 5 | 4.9931 | 0.2554 | 0.5263 |
|  | 6 | 5.0762 | 0.3318 | 0.6627 |
|  | Value of the index | 5.9294 | 0.5215 | 0.9764 |
| 48 h | 2 | 5.2383 | 0.5013 | 0.8630 |
|  | 3 | **5.8720** | 0.3954 | **0.9633** |
|  | 4 | 5.3862 | **0.5023** | 0.8757 |
|  | 5 | 4.5027 | 0.3341 | 0.5675 |
|  | 6 | 5.1466 | 0.3819 | 0.7898 |
|  | Value of the index | 5.872 | 0.5023 | 0.9633 |

| **Source** | **Metabolite** | **Dim.1** | **Dim.2** | **Dim.3** | **Dim.4** | **Dim.5** |
| --- | --- | --- | --- | --- | --- | --- |
| Carbon | L-Asparagine | 0.1486 | 0.0455 | 0.0014 | **0.8279** | 0.005 |
| Carbon | Methyl Pyruvate | 0.0026 | 7.0E-04 | 0.0964 | **0.8391** | 0.0988 |
| Carbon | Amygdalin | 0.0374 | 0.1885 | **0.8399** | 0.3662 | 0.0024 |
| Carbon | L-Lysine | 0.8907 | 0.0309 | 0.0035 | **0.8067** | 0.017 |
| Carbon | N-Acetyl-D-Galactosamine | 0.0868 | 0.2079 | 0.1164 | **0.8793** | 0.0012 |
| Nitrogen | Ala-Gly | 0 | 0.1661 | **0.8886** | **0.8203** | 0.1026 |
| Nitrogen | α-Amino-N-Valeric Acid | 0.1534 | 0.2229 | **0.8062** | 2.0E-04 | 0.0383 |
| Nitrogen | Ammonia | 0.1692 | 0.2191 | 0.1207 | **0.8586** | 0.0017 |
| Nitrogen | D, L-α-Amino-N-Butyric Acid | 0.2528 | 0.0899 | **0.8481** | 0.1407 | 0.0341 |
| Nitrogen | L-Phenylalanine | 0.3305 | 0.1346 | **0.8512** | 0.0402 | 0.0302 |
| Nitrogen | N-Acetyl-D-Galactosamine | 0.054 | 0.3468 | 0.101 | **0.8869** | 0.0262 |
| Nitrogen | Nitrate | 0.0302 | 0.0976 | **0.8868** | 5.0E-04 | 7.0E-04 |
| Nitrogen | Urea | 0.1365 | 0.32 | **0.8525** | 0.1138 | 6.0E-04 |
| Nitrogen | Uridine | 0.1445 | 0.3867 | **0.8026** | 4.0E-04 | 0.0062 |
| P and S | L-Cysteine | 0.452 | 0.2085 | 0.006 | **0.8033** | 0.2268 |
| P and S | Pyrophosphate | 0.4212 | 0.0272 | 0.0401 | **0.8473** | 0 |
| Nutrient | L-Citrulline | 0.6421 | 0.0952 | 0.0338 | **0.8239** | 0.0055 |
| Nutrient | Tween 80 | 0.0062 | 0.0501 | **0.8498** | 0.0087 | 0.0397 |

Supplementary Table 2A. Squared cosine values of variables included in the multiple factor analysis. Metabolites with *cos^2^* above 0.8 for Dimension 3 and 4 were considered to potentially promote respiratory activity, because these dimensions described beneficial bacteria with similar respiratory activities at 24 h.

Supplementary Table 2B. Squared cosine values of variables included in the multiple factor analysis. Dimension 3 described pathobionts, while dimensions 4 and 5 include and beneficial bacteria with similar respiratory activities at 48 h. Thus, metabolites with *cos^2^* above 0.8 for Dimension 3, 4 and 5 were considered to potentially promote respiratory activity.

| **Source** | **Metabolite** | **Dim.1** | **Dim.2** | **Dim.3** | **Dim.4** | **Dim.5** |
| --- | --- | --- | --- | --- | --- | --- |
| Carbon | Glycyl-L-Aspartic Acid | 5.0E-04 | 0.1958 | 0.0834 | **0.8498** | 0.0095 |
| Carbon | Glycyl-L-Glutamic Acid | 0.0067 | 0.3806 | 0.0874 | **0.8452** | 0.0267 |
| Carbon | Glycyl-L-Proline | 0.0515 | 0.031 | 0.0075 | **0.8663** | 0.1222 |
| Carbon | L-Alanyl-Glycine | 1.0E-04 | 5.0E-04 | **0.8536** | **0.8325** | 0.0047 |
| Carbon | L-Glutamine | 0.0043 | 0.0179 | 0.0335 | **0.8756** | 0.0393 |
| Carbon | γ-Hydroxy Butyric Acid | 0.327 | 0.0052 | **0.806** | 0.1468 | 0.0011 |
| Carbon | Gelatin | 4.0E-04 | 0.3386 | 0.1976 | **0.8341** | 0.0139 |
| Carbon | L-Isoleucine | 0.0641 | 0.1436 | **0.8383** | **0.8567** | 1.0E-04 |
| Carbon | L-Leucine | 0.1276 | 0.1227 | 0.2559 | **0.8273** | 0.0122 |
| Carbon | L-Methionine | 0.0985 | 0.059 | **0.811** | 0.1819 | 0.0438 |
| Carbon | L-Valine | 0.0106 | 0.2356 | **0.8315** | **0.8356** | 0.0033 |
| Nitrogen | Ala-Gly | 0.0187 | 0.3625 | **0.8811** | 0.1661 | 0.0119 |
| Nitrogen | Gly-Asn | 0.0147 | 0.4622 | 0.0867 | **0.8401** | 0.0052 |
| P and S | 2-Hydroxyethane Sulfonic Acid | 0.4378 | 0.0869 | 0.0827 | **0.8155** | 0.0135 |
| P and S | Adenosine-3',5'-cyclic monophosphate | 0.0288 | 0.0191 | **0.8588** | 0.3007 | 0.0017 |
| P and S | D-Glucose-1-Phosphate | 0.1661 | 0.2105 | **0.8126** | 0.1751 | 0.0414 |
| P and S | L-Cysteine Sulfinic Acid | 0.1443 | 0.1453 | 0.038 | **0.856** | **0.8061** |
| P and S | N-Acetyl-L-Cysteine | 0.2854 | 0.0409 | 0 | **0.8796** | 0.0913 |
| P and S | O-Phospho-D-Tyrosine | 0.3377 | 0.0413 | **0.8163** | **0.8048** | 0.0485 |
| P and S | Taurine | 0.2587 | 0.1666 | 0.0772 | **0.8145** | 0.0816 |
